# How does moulage contribute to medical students’ perceived engagement in simulation? A mixed-methods pilot study

**DOI:** 10.1186/s41077-020-00142-0

**Published:** 2020-08-26

**Authors:** Jessica B. Stokes-Parish, Robbert Duvivier, Brian Jolly

**Affiliations:** 1 School of Medicine & Public Health, University of Newcastle, Callaghan, New South Wales Australia; 2 Center for Educational Development and Research in Health Sciences, University Medical Center Groningen, Groningen, The Netherlands; 3 School of Rural Medicine, University of New England, Armidale, New South Wales Australia

**Keywords:** Moulage, Engagement, Instructional design, Medical education, Realism

## Abstract

**Introduction:**

Moulage is used frequently in simulation, with emerging evidence for its use in fields such as paramedicine, radiography and dermatology. It is argued that moulage adds to realism in simulation, although recent work highlighted the ambiguity of moulage practice in simulation. In the absence of knowledge, this study sought to explore the impact of highly authentic moulage on engagement in simulation.

**Methods:**

We conducted a randomised mixed-methods study exploring undergraduate medical students’ perception of engagement in relation to the authenticity moulage. Participants were randomised to one of three groups: control (no moulage, narrative only), low authenticity (LowAuth) or high authenticity (HighAuth). Measures included self-report of engagement, the Immersion Scale Reporting Instrument (ISRI), omission of treatment actions, time-to-treat and self-report of authenticity. In combination with these objective measures, we utilised the Stimulated Recall (SR) technique to conduct interviews immediately following the simulation.

**Results:**

A total of 33 medical students participated in the study. There was no statistically significant difference between groups on the overall ISRI score. There were statistically significant results between groups on the self-reported engagement measure, and on the treatment actions, time-to-treat measures and the rating of authenticity. Four primary themes ((1) the rules of simulation, (2) believability, (3) consistency of presentation, (4) personal knowledge ) were extracted from the interview analysis, with a further 9 subthemes identified ((1) awareness of simulating, (2) making sense of the context (3) hidden agendas, (4) between two places, (5) dismissing, (6) person centredness, (7) missing information (8) level of training (9) previous experiences).

**Conclusions:**

Students rate moulage authenticity highly in simulations. The use of high-authenticity moulage impacts on their prioritisation and task completion. Although the slower performance in the HighAuth group did not have impact on simulated treatment outcomes, highly authentic moulage may be a stronger predictor of performance. Highly authentic moulage is preferable on the basis of optimising learning conditions.

## Introduction

Engagement in simulation is described as a key to success; if a participant is engaged, the learning/simulation must have “worked”. Grounded in the notion of active learning theories such as experiential learning and constructivism, engaged learners “construct knowledge from experience, meaning interpretation and having interactions with peers” (Hung et al. 2006). But what is engagement? In gaming, engagement is described as being associated with qualities that pull people in [1]. Hung et al. (2006) describe engaged learning as “authentic”, whereby learners are able to problem-solve, make choices and interact with peers and instructors [2]. Simulation incorporates this in the very nature of its delivery—participants are given a case they must work through, often in a group. In simulation, the word engagement is often interchanged with the word “immersion”. Immersion is the “subjective impression that one is participating in a comprehensive, realistic experience” [3]. This highlights the individual part of being able to suspend disbelief to participate actively in the simulation. This concept of engagement is echoed by many authors [4–6], yet there has been little discussion on what engagement means in the context of simulation. Indeed, Padgett et al. raise this in a critical narrative review of the definition of engagement in simulation, agreeing that the term engagement is used loosely and without clear definition [7]. In their terms: “Learner engagement is a context-dependent state of dedicated focus towards a task wherein the learner is involved cognitively, behaviourally, and emotionally” [7]. However, Padgett et al. do not explore gaming literature, the concept of suspending disbelief or the likeness between immersion and engagement [7]. For the purpose of this study, we have defined engagement as

*the state in which the participant is observed to be actively interacting with the simulation as if it were real*.

With the opposite being true of disengagement, the participant is unable to interact as if it were real.

Experts posit strategies to increase engagement through realism. Moulage is increasingly described as a way to increase realism in simulation. Defined as “the use of special effects makeup techniques to simulate illnesses, bruises, bleeding, wounds or other effects to a manikin or simulated patient, acting as visual and tactile cues for the learner” [8], moulage is used at varying levels in simulation scenarios. Since the publication of our commentary, [9], a number of studies have been published to explore its use and benefit in simulation. One such study by Mills et al. (2018) explored how immersion is influenced by the use of moulage, resulting in a significant difference between control and experimental groups where no moulage versus moulage was tested in a study on paramedicine students [10]. In this study, participants were randomised to two groups (no moulage or moulage) and researchers measured task immersion, eye-tracking and interviews. Moulage is gaining attention in other fields, such as radiology [11], where it has not been explored before, whilst areas like dermatology continue to research the use of moulage as a teaching method for melanoma identification [12, 13]. In other fields of simulation, such as military or defence training, highly authentic moulage is often a de facto inclusion that is regarded highly important [14].

We have identified elsewhere the need to explore how moulage contributes to simulation, as opposed to a sort of de facto inclusion in simulation instructional design. We propose that moulage fits in the domains of realism suggested by Dieckmann et al. [5]. That is, moulage is physical (the moulage appears real), semantic (moulage is conceptually believable—if A occurs, B will happen, so therefore I engage) and phenomenal (I emotionally engage with the case because moulage enhances first impressions). However, we do not understand precisely how moulage fits within this framework. A moulage should be believable, make sense to the viewer and not in a contradictory manner. We hypothesise that if a moulaged wound does not match the narrative or if it was portrayed inaccurately, this could disrupt the participants’ engagement, potentially influencing engagement in learning activity. This hypothesis is supported by literature where episodes of disengagement occurred in simulations where the narrative or setting were not plausible or factual [15].

The aims of this study were to answer the following questions:

1. How does the use of moulage authenticity impact on engagement of participants in a healthcare simulation?
2. What are stakeholders perceptions of the value of high and low-authenticity moulage compared to none in the educational process?

To answer these questions, we had the following hypotheses:

- Hypothesis 1: Highly authentic moulage causes greater engagement in simulation participants
- Hypothesis 2: Poorly authentic moulage causes disengagement in simulation participants

In the following sections, we describe the methods and study design for this work, before moving on to the results.

## Methods

### Participants

We recruited participants from the final 2 years of the undergraduate medical degree (5 years) at the University of Newcastle in Australia. Students were eligible to participate in the study if they had participated in simulations previously as a part of their degree. Students were not eligible to participate if they had no previous experience participating in highly immersive simulations or if they wore glasses (due to the eye-tracking component of the study, contacts were allowed).

Based on power calculations from previous studies [16] and the size of a useful or meaningful difference, we identified that a sample of 21 participants in the control group and 18 each in the experimental groups would be needed to detect an effect size of 0.8 with a power of 90% between control and experimental wings. A slightly larger sample size (*n* = 23) was required to detect differences of the same magnitude (0.8) between the two experimental conditions. Meta-analysis of over 1500 educational interventions suggest that the average effect size for any intervention is 0.4, so effect sizes greater than 0.4 were identified as worth pursuing and reliably detecting [17].

Recruitment took place via lectures and online postings on the course website. Flyers were placed in the student common rooms, library and student-teaching areas. The invitation included information regarding the duration and location of the study, and the study aims. After a student expressed interest, they were sent the full Participant Information Statement and invited to book in a session at the simulated laboratory. Participants were randomised into control and experimental groups to participate in a trauma simulation. The control group was narrative case only, whilst experimental groups were both narrative case and moulage. That narrative case and moulage are described in more detail below. The experimental groups were further randomised to either highly authentic or inauthentic moulage. All data was collected in late semester 2 of 2017 and 2018. The study protocol was approved by the University of Newcastle Human Research Ethics Committee (H-2017-0214).

### Randomisation

Participants were given a unique ID code using randomizer.com. The Research Assistant generated the random codes independent of the Chief Investigator (JSP) and allocated the codes at random to the participants. The Chief Investigator was only aware of the randomisation on the day of the study. Participants were told that they would be randomised to one of the three groups, but were blind to the group they were allocated until the simulation commenced.

### Orientation to simulation and study

Participants were given a standard simulation orientation to the location, including covering the fiction contract (the process in which the participant agrees to interact in the simulation within the set rules of simulation), confidentiality agreement and ground rules for participation in simulation, as per the International Nursing Association for Clinical Simulation and Learning (INACSL) Standards for Simulation [18] as well as an outline of how the study would flow. At this point, the participants signed consent to participate. Following this, eye-tracking equipment was applied and calibrated (the results of the eye-tracking study will be reported in a later paper). The participant was then familiarised with the manikin, props and surrounding equipment. This included talking to the manikin, conducting a physical examination and meeting the confederate. The participant was then invited to sit outside the simulation room and read the scenario brief. This entire process was completed by the Research Assistant.

## Materials

### Scenario

**Fig. 1:**
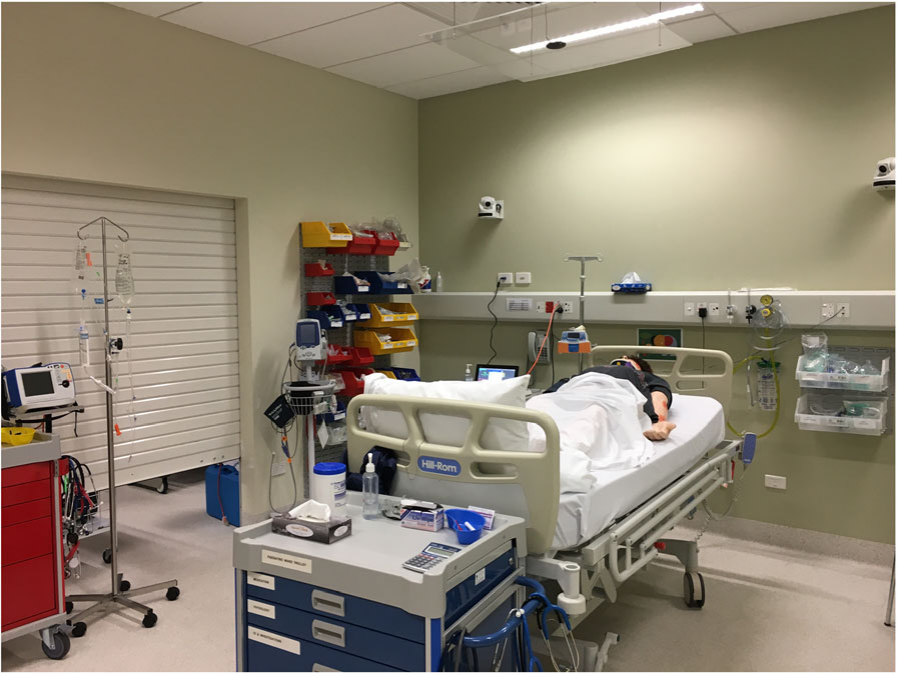
Simulation room

The scenario chosen was previously assessed for content validity and a peer-reviewed trauma scenario [19]. We chose to use only one scenario due to the resource-intensive nature of the study. The selected scenario was a male who was brought in by ambulance to the local Emergency Department (ED) following a mountain bike accident. Participants were given an ED Admissions sheet outlining the presenting complaint and were then called in to the scenario by the confederate Endorsed Enrolled Nurse (EEN) to come and review the new patient in ED. All study conditions took place in the Chameleon Simulation Centre in a well-lit room, quiet and devoid of extraneous props. Each participant completed the scenario individually. A confederate EEN was in the room providing narrative cues and assisting with nursing tasks. The simulated emergency room was set up to replicate local emergency department rooms. The room consisted of a bed, oxygen/air outlets, suction, oxygen delivery devices, emergency resuscitation trolley, bed, intravenous (IV) fluids pump, observations monitor, standard equipment trolley and a bed. The stock and equipment trolleys included mock medications, fluids, wound dressing supplies and various other medical equipment relevant to trauma scenarios (Fig. 1).

The confederate was given training prior to the commencement of the study. They were taught to troubleshoot technical issues within the scenario, instructed on how to respond to the students and were instructed to not prompt action or correct clinical decisions that they perceived as errors. The confederate was equipped with a one-way ear piece in which the scenario manager could feed information if required.

### Variable

The only difference between the groups was the appearance, i.e. moulage or no moulage.

In the control group, the manikin had no moulage applied. Instead, the confederate would give the participant a verbal cue describing the areas of injury (e.g. “there are some grazes and cuts on the face”, and “he has a bruise on his stomach” and “there is a laceration and grazing to the left arm”). In the low- and high-authenticity groups, the confederate only gave verbal cues if the participant requested further information about the wounds (e.g. "no, there is no active bleeding”).

The authenticity of moulage was rated by independent clinicians from a variety of specialties using the Moulage Authenticity Rating Scale (MARS) [20] (Full makeup application description in the Appendix). Following reliability testing, we compressed the elements of the MARS into two categories—the Physical and Cognitive Scales of Authenticity. We completed a Comparison of Scale Means utilising a one-way analysis of variance (ANOVA) for 3-group comparisons and *t* tests for 2-group comparisons. The results are detailed in Table 1.

**Table 1:** Expert rating of authenticity

Wound		Control Mean (*n*)	LowAuth Mean (*n*)	HighAuth Mean (*n*)	Statistical significance* (*p* < 0.05)
Arm	Physical	11.2 (6)	17.3 (4)	16.0 (8)	*p* 0.039*
	Cognitive	9.9	23.8	12.6	*p* 0.000*
	All elements	21.2	41.0	28.6	*p* 0.000*
Abdominal	Physical	10.5 (5)	15.0 (7)	NA	p 0.119
	Cognitive	13.5	18.6		p 0.213
	All	24.0	33.6		p 0.086
Facial	Physical	11.5 (5)	16.1 (6)	15.5 (9)	p 0.116
	Cognitive	19.6	11.8	16.4	p 0.026*
	All	31.1	28.0	31.9	p 0.563

## Measures

### Immersion

Video footage of the simulation was reviewed to identify episodes of engagement or disengagement. Using the Immersion Score Rating Instrument (IRSI) [4], the footage was reviewed by JSP at a later date and the results were then discussed with the other authors. The ISRI is a tool to measure participant immersion within the simulation. Despite the use of the word immersion, we interpreted the authors’ intent as to measure engagement. Although these are subjective measures, we considered them appropriate for the study at hand, particularly since the engagement of participants was measured by additional outcomes—such as eye-tracking glasses, engagement self-report and stimulated recall interviews.

### Clinical markers

Participants’ performance was assessed by means of clinical performance and time-to-treat (that is, how long it took them to achieve expected actions). The expected clinical performance included physical assessment, administration of intravenous fluids, ordering an ultrasound, administration of oxygen and was verified by expert clinicians elsewhere [19]. This data was collected through the Laerdal LLEAP® program and through observational measures. JSP extracted the Laerdal Scenario actions file and then observed the videos and noted actions taken by participants, including timestamp. These codes were discussed with the other authors throughout the coding process to ensure representativeness. These observations were compared across groups, by means of difference in time-to-treat and omission of actions.

### Self-report measures

Immediately following the scenario, participants completed a survey to report their perceived engagement with the scenario and the perceived reality of the visual cues (face and content validity). This survey was an adaptation of the survey used by Pywell et al. [21]. The adaptation included additional questions regarding perceived engagement and refocusing the questions on face and content validity to be trauma based (see Appendix 3).

In addition to this, participants rated the authenticity of moulage using the Moulage Authenticity Rating Scale (MARS) [20]. Both self-report measures were compared across groups to determine differences, if any.

### Interviews

Participants were interviewed following the simulation using video-stimulated recall techniques [22]. This method was selected to explore how the moulage authenticity impacts on participant engagement (H1) and their perceptions of high and low-authenticity moulage (H2). Stimulated recall techniques are recommended to enhance recall of events and to complement eye-tracking methodologies, aligning thoughts with action [23]. The interview questions were structured with a general framework, however, were flexible enough to explore areas of deeper focus. The central themes of the interview focused on engagement and moulage. The guide for interviews can be found in Appendix 2. The interviews were audiotaped and transcribed verbatim by a professional academic transcription service. Drawing from Grounded Theory techniques, the interviews were analysed using a four-phase process. The first phase was familiarisation with the literature (reading transcripts and listening to the audio recording), followed by an initial code, then a categorical coding process, and, finally, making meaning. Using a manual process, JSP coded line by line, noting sentences and phrases that described the underlying meaning. This continued until saturation was reached, at which point they were categorised in to groups. Throughout this process, JSP consulted with the other authors on the coding (BJ, RD), took memos and reflective notes to synthesise the evidence and gradually build meaning.

### Statistics

IBM® Statistical Software Package for Social Science (SPSS v. 23) was used for all statistical comparisons. Statistical significance was defined as a value of 0.05. We used one-way ANOVA to compare groups, dependent on the level of measurement of the data (ISRI), time-to-action, MARS, self-reported engagement and used further post hoc tests (Tukey’s) where appropriate to determine differences between the three groups. We performed chi-squared tests with Fisher’s exact to compare the clinical actions completed.

## Results

A total of 33 undergraduate medical students were recruited in the latter half of semester 2 in 2017 and 2018. Of these participants, 15 were year 4 medical students and 18 were year 5. The participants had good exposure to simulation-based education, including Advanced Life Support training. Twenty-two (66%) of the participants were females and 11 were males (33%). Nine were randomised to the control group, 13 to low authenticity (LowAuth) and 10 to high authenticity (HighAuth).

In this section, we break down the relevant results in their measurement groups in the same categories as the methods description. The full data can be seen in the supplementary file (Appendix 1).

### Clinical actions

#### Clinical actions completed

Data were available from 32 of the 33 participants. Data from one participant were lost due to a technical glitch. Groups were compared on the following indices: whether they completed hand hygiene at any point during the encounter, requested an ultrasound, ordered IV fluids, exposed the abdomen, examined the abdomen, called for help and investigated or treated the injury cues. We performed chi-squared statistics comparing whether groups completed expected actions.

In these clinical actions, there was a trend of completing clinical actions in the high-authenticity (HighAuth) moulage group as compared to other groups (neurological observations, *p* = .04) and a trend to complete an abdominal palpation with the low-authenticity moulage (LowAuth) group (*p* = .03). When comparing combinations, there was a statistically significant difference in the control/LowAuth group to conduct an abdominal palpation (*p* = .02). Differences between all other indices were not significant. See Table 2 for a visual representation of what clinical actions were completed.

**Table 2:** Clinical actions completed by participant

	C ***n*** = 9 (% in group)	LowAuth ***n*** = 13 (% in group)	HighAuth ***n*** = 10 (% in group)
**Hand hygiene at commencement of scenario**^**a**^	3 (33%)	6 (46%)	6 (60%)
**Gloves**	1 (11%)	2 (15%)	5 (50%)
**Abdominal ultrasound**	2 (22%)	6 (46%)	5 (50%)
**Intravenous fluids**	8 (89%)	12 (92%)	10 (100%)
**Neuro observations**^**b**^	5 (56%)	1 (1%)	4 (40%)
**Pathology**	5 (56%)	6 (46%)	5 (50%)
**Abdominal palp**^**b**^	8 (89%)	13 (100%)	6 (60%)
**Called for help**	6 (67%)	9 (70%)	6 (60%)
**X-ray**	3 (33%)	6 (46%)	4 (40%)
**Investigated injury cues**	5 (56%)	7 (54%)	8 (80%)
**Treated injury cues**	1 (11%)	2 (15%)	3 (30%)

^a^Participants did not complete any further hand hygiene throughout the scenario

^b^Significant differences chi-square between the 3 groups

#### Time-to-treat

To determine any differences between groups on the time-to-treat, we conducted one-way ANOVA. In the instance that a participant did not complete the action, we treated them as if they would have taken the longest time to complete the action. We compared the three groups by one-way ANOVA and further post hoc tests (Tukey’s HD) where applicable.

The full analysis can be viewed in Table 3. In exposing the abdomen, the LowAuth group took the longest (115.92s, SD 97.82) and the control group the shortest (69.77 s, SD 43.55). Participants in the HighAuth group took the longest to call for help (245.1 s, SD 97.71), while the LowAuth group called for help the quickest (197.15 s, SD 95.30). When requesting an ultrasound, the HighAuth group ordered it the quickest (193 s, SD 82.28) and the control group the slowest (242.66 s, SD 38.44). The HighAuth group took the longest to order intravenous fluids (174.9 s, SD 88), whilst the LowAuth group were the quickest (112.41 s, SD 49.69). There were no statistically significant differences between the groups.

**Table 3:** Mean times to action

		*N*	Mean	Std. deviation	Std. error	95% confidence interval for mean	
						Lower bound	Upper bound
Hand hygiene	Control	9	71.4	36.1	12.0	43.6	99.2
	LowAuth	13	51.7	43.0	11.9	25.7	77.7
	HighAuth	10	41.3	42.1	13.3	11.1	71.4
Exposes abdomen	Control	9	69.7	43.5	14.5	36.2	103.2
	LowAuth	13	115.9	97.8	27.1	56.8	175.0
	HighAuth	10	72.4	51.4	16.2	35.6	109.1
Calls for help	Control	9	211.3	89.8	29.9	142.2	280.3
	LowAuth	13	197.1	95.3	26.4	139.5	254.7
	HighAuth	10	245.1	97.7	30.9	175.1	315.0
Orders fast scan	Control	9	242.6	38.4	12.8	213.1	272.2
	LowAuth	13	220.7	60.0	16.6	184.4	257.0
	HighAuth	10	193.0	82.2	26.0	134.1	251.8
Inspects injuries	Control	9	214.0	99.4	33.1	137.5	290.4
	LowAuth	13	252.0	95.4	26.4	194.4	309.7
	HighAuth	10	200.0	82.1	25.9	141.2	258.7

### Immersion

We ran a one-way ANOVA by group of the ISRI (Table 4), where the mean score across all experimental groups was 38.59 (SD 14.45). There was no statistically significant difference between the experimental groups. We drilled down further to explore if there was a difference between undergraduate year and gender. There was no statistically significant result between year 4 and 5 students or between genders (M/F). In a *t* test (with Levene’s test for equality of variances) comparison of moulage (combined LowAuth and HighAuth) versus no moulage (control), there was no statistically significant difference (Table 5). Despite this lack of significance, when observing the scatterplot representation of the means, HighAuth had less variability in immersion scores (see Appendix 1) as compared to both the control and LowAuth group.

**Table 4:** One-way ANOVA of ISRI

	*N*	Mean	Std. deviation	Std. error	95% confidence interval for mean	
					Lower bound	Upper bound
Control	9	33.4	17.1	5.7	20.3	46.6
Experimental group 1	13	43.2	15.9	4.4	33.5	52.8
Experimental group 2	10	37.4	8.2	2.5	31.4	43.2

**Table 5:** *t* test comparison of ISRI scores

Item (***n***)	Mean (SD)
Year 4 (14)	34.2 (14.9)
Year 5 (18)	42 (13.6)
Male (10)	38.3 (12.5)
Female (22)	38.7 (15.6)
Moulage (9)	33.4 (17.1)
No moulage (23)	40.6 (13.2)

### Self-report measures

#### Engagement survey

In all groups, the participants felt they were engaged. The participants rated moulage as important in all groups and felt that the lack of moulage did contribute to disengagement (*p* = 0.02). When exploring the realism of the scenario, the participants in the HighAuth group rated the realism higher (*p* = 0.01) and as representative of trauma compared to the other groups (*p* = 0.00). The moulage contributed to the participant’s ability to treat the simulation as if it were real and made them feel like they were in a real trauma situation (*p* = 0.01). The presence of moulage in both the LowAuth and HighAuth groups contributed to a positive training experience (*p* = 0.03). Full results are presented in Appendix 1.

#### Moulage authenticity rating

When comparing participants’ ratings on the authenticity of moulage, there were statistically significant differences between groups across the scales. The ANOVA identified differences between the groups in the physical, cognitive and all elements scales. Overall, the participants rated the moulage as most authentic in the HighAuth group when rating the elements individually (position, *p* = 0.02; detail, *p* = 0.00; likeness to real world; *p* = 0.00; colour, *p* = 0.00; size, *p* = 0.04) and in the global rating of authenticity (*p* = 0.00). When comparing the physical and cognitive scales where there was little difference between the LowAuth and HighAuth group. In post hoc analysis of the physical scale, there was a statistically significant difference between control and HighAuth (*p* = 0.00) and control and LowAuth (*p* = 0.02). In post hoc analysis of the cognitive scale, there was a statistically significant difference between control and HighAuth (*p* = 0.00) and control and LowAuth (*p* = 0.00). In the all elements scale, there was statistical significance between groups (p 0.00) and within control vs HighAuth (*p* 0.00), but not control vs LowAuth or LowAuth vs HighAuth. The use of moulage was strongly correlated with a rating of authenticity, as opposed to no moulage. The full analyses of results are accessible in Appendix 1.

### Interviews

#### Thematic summary

Four primary themes emerged from the participant interviews, including (1) the rules of simulation, (2) believability, (3) consistency of presentation and (4) personal knowledge. Within these themes, subthemes appeared: (1) awareness of simulating, (2) making sense of the context (3) hidden agendas, (4) between two places, (5) dismissing, (6) person centred-ness, (7) missing information (8) level of training and (9) previous experiences.

#### The rules of simulation

Participants described the process of determining the rules of simulation and learning how to settle into simulation. They expressed challenges determining if what they were doing was an actual part of the simulation or a condition of the simulation. Participants described instances of attempting to progress through the simulation whereby they needed to make sense of the context of simulation, determine if there were hidden agendas; they demonstrated an experience of being between two places to make meaning of the rules of simulation. That is, they were aware they were simulating, yet they were mentally processing the conditions of simulation versus reality at the same time.

#### Awareness of “simulating”

The more participants were aware of the simulation, the less engaged they were; meaning they were not necessarily engaged in learning, but more focussed on determining the rules of simulation. For example, participant 22 (control group) expressed “*as soon as I looked and then saw it was like crystal clean…it just like kind of pulls you back in, okay it’s a simulation*”. Participant 58 (control group) said regarding the lack of moulage and its contribution to engagement “*the engagement in believing it was real was less so. Like I took it as oh this is a simulation now, I’m going to be doing a simulation*…”.

They identified that they had a constant background awareness that they were simulating, at varying degrees, depending on the level of authenticity presented. Participants described this type of engagement as more of a check-box activity, a “*going through the motions*” as opposed to meaningful learning activity.

*“I guess at the back of my mind there's always this idea of that this is just a simulation. Yeah. I think I wasn't - I don't know, I think I wasn't having that feeling, oh okay this is real, I really have to do something about this patient, yeah. It was more like going through the motions”* (participant 20, experimental group)

#### Making sense of the context

Participants described attempting to make sense of the simulated conditions by verifying cues presented, searching for additional cues (that they otherwise would not look for in a real patient) and questioning their own judgements. Participant 14 (control group) identified feeling confused – *“…is this the site or am I just imagining it…I disengage and went into my own thoughts because…I wasn’t 100 percent sure that what I was…an issue*”. This confusion was echoed by participant 39 (LowAuth), they said “…*you can’t visualise so you don’t know whether he is supposed to have a bruise or whether he really doesn’t have any bruise. So you have to assume…*”.

#### Hidden agendas

Participants felt there were hidden purposes to the simulation itself. In some instances, they described taking the confederates’ cues (instead of visually presented) as if to mean there was importance to the cue, leading them to pursue that particular path, participant 14 says “*oh okay, I’m missing something again*”. In their mind, if a confederate voiced a cue, there was hidden meaning behind it—“*they’re telling me about it so it must be the main important thing*” (participant 50, HighAuth). Participants expressed an expectation that there was something going to happen—the patient would “crash” and require emergency treatment, mostly because prior scenarios they were involved in went down the path of cardiopulmonary resuscitation. For example, participant 12 (LowAuth) noted “*I thought you’re going to make him crash on me. It’s like a classic*”.

#### Between two places

This subtheme describes the degree to which participants were aware that they were “in” a simulation. Multiple participants described “stepping in” and “stepping out” of the simulation, for example, participant 12 (LowAuth) says: “[I]..*have to switch out of the scenario to check things out. In real life you can either see it’s happening or it’s not*”. When they are fully engaged, participants are able to progress through the simulation and engage with the cues presented; when they are confused about the cues presented or unsure of the believability, participants described needing to “step out” to verify the conditions of the simulation – “*I did disengage in the sense that I had to then pull myself out of it and thought – all right, let’s just evaluate what’s happened, rather than keep rolling on*” (participant 10, LowAuth).

### Believability

Throughout the interview analysis, participants repeatedly described a desire or need to be able to “believe what they see”. They identified that they wanted visual cues to be convincing as they felt the cues contributed to overall engagement and sense of reality. Participants expressed that the lack of reality created confusion, leading them to not take the scenario seriously. The students identified that this is a crucial aspect for their learning, as they felt there was no point in a simulation if it did not allow them to practice an assessment in an authentic way. Participant 40 (HighAuth) describes, “*they look human-like…it sets you up very well for a clinical scenario…*” and participant 50 (HighAuth) highlights “*we’re trained to always be looking at the whole page …looking for every little detail about the patient to see what you can glean about their clinical situation”.* Believable moulage encouraged them to treat the scenario as if it were real and to physically complete actions instead of pretending to.

#### Dismissing

A consistent theme in the interviews was the idea of dismissing or ignoring the cues if they were delivered verbally (C) or represented poorly (LowAuth). Participants in LowAuth expressed they viewed and they assumed the moulage was unimportant due to the unidimensional aspect.

On the inclusion of moulage, participant 40 (HighAuth) says *“it just gives it a good indication of where they've been hit which you - we don't have otherwise in these trauma cases that we get… otherwise you just have to ask everything. You don't know what he has and what he doesn't have unless you're specifically told…you'd never ask that or you wouldn't normally ask that in a normal clinical situation because you can see it”.* When the reasons for dismissing where explore further, participants described feeling overloaded with information, causing them to forget – “*I missed that cue. I completely forgot that the nurse …said tha*t” (participant 21, LowAuth).

#### Person-centredness

Participants described the impact of authentic moulage in terms of how they approached the patient. For example, they valued engaging with the patient verbally, and the moulage provided a trigger to remind them to take the simulation seriously; in their view, the interaction became more patient-focused because of the presence of moulage. For example, participant 14 (control) says “*I snapped out of the situation again…thinking more in terms of a manikin than a human”*.

### Consistency of presentation

Participants valued the consistency of presentation of visual cues and how the cues interacted with the rest of the story. They repeatedly described that the combined cues contributed to how well they engaged in simulation. It was not one single aspect that contributed more.

#### Missing information

Participants described missing information as a trigger for disengaging from the simulation. In these instances, they described being reminded that it was a simulation, and that there were limitations. Additionally, they felt that missing information was a limitation to learning how to assess patients; in their view, authenticity forced independent thinking and assisted them to understand how they might behave in real life. Participant 1 (HighAuth) says “*the moulage is good and it’s showing what it’s meant to…that would be really good, but if it’s just like a sticker or something that says ‘blood here’, then that might detract from the situation because I’m like I’ll have to ask heaps of questions about that sort of thing”.*

### Personal knowledge

Personal knowledge was described as a cause of disengagement in the simulation. This was two-dimensional: the level of clinical training the participant had and the previous experiences in simulation.

#### Level of training

Participants described being unable to progress in the simulation if they got to a point at which they had no experience. For example, deciding what treatment decision would come next, participant 13 (HighAuth) described feeling at the limit of what they could do after attempting to manage the blood pressure: “*I disengaged a little bit here but this is just my lack of knowledge, rather than the actual situation itself*”.

#### Previous experiences

Beyond this, the participants repeatedly referred to their previous experiences in simulation and how that influenced their interaction with the moulage. Participants described confusion between conditions of simulations and simulated assessments. For example, participant 50 (HighAuth) described simulated formative assessments where instead of *doing* the clinical activity, they *talked* about what they would do – “*I’m used to OSCEs* [Objective Structured Clinical Examinations]…*I say everything out loud…It’s the worst, it’s so bad clinically*”. In addition to this, participants described the lack of authenticity in previous simulations (non-OSCE type simulations) lead them to treat future simulations with less believability.

*“I’ve done previous simulations before where it’s like you’re very much, you look at someone and you say what are the obs? How is the heart rate, kind of thing and you just go from there? And I sort of just went back into that … as opposed to actively searching for wounds or actively feeling the pulse…”* (participant 58, control)

## Discussion

The study described sought to explore the potential relationship between the authenticity of moulage and participant engagement in undergraduate medical students. To our knowledge, this is the first study of its kind in any health professions field. In this discussion, we link the results described above with the hypotheses presented in the introduction and present the potential links to simulation practice in medical education.

We predicted that higher levels of authenticity would improve participant engagement in simulation (H1). This hypothesis was supported by the self-report results, whereby students rated highly authentic moulage as less likely to contribute to episodes of disengagement and lack of moulage was likely to contribute to disengagement (H2). However, participants in all three groups agreed that they felt engaged throughout the scenario, which makes H1 less plausible. This finding was supported by the results of the ISRI, in which there were no significant differences between groups. We are unsure if this is due to the small study size or a true representation. In the scatterplot representation of the ISRI scores, the control group had more widely distributed responses; the pattern of HighAuth results might suggest more consistent engagement with the inclusion of authentic moulage than the other groups. These findings of the authenticity rating scale (MARS) also suggested that some moulage, as opposed to authentic moulage, was sufficient for engagement (further making H1 less plausible). One explanation for the ability to engage regardless of authenticity might be that medical students are known to have high levels of motivation—they may already have motivation to engage within a simulation [24]. This was echoed in the interviews with participants, where they talked about an ability to just continue on and reset their engagement. However, participants also discussed constantly searching for something to engage with, either by responding to visual cues or by way of dismissing what they were unable to reconcile within the simulation. This could describe a sort of disengagement, supporting H2. However, perhaps the ability to engage despite the level of authenticity is as a result of extrinsic motivator factors, whereby the individual is motivated by pressure of others (such as the presence of a confederate nurse or the continual flow of the simulation) or perhaps this is what Padgett et al. refer to in being “focused towards a task” [7].

A secondary aspect of the study was to explore students’ perceptions of the authenticity of moulage in simulation. All three groups identified that the authenticity of moulage is important in simulation, and participants in the control or LowAuth moulage groups did not perceive their encounter to be a realistic representation of a trauma scenario in the survey. However, their limited exposure to simulation and real trauma may have limited their ability to truly rate this. From an opposing perspective, perhaps this reinforces the importance of accurate moulage portrayal for inexperienced clinicians. Extending on this idea of perceiving authenticity, the participants highlighted the impact of previous simulation authenticity and design; that is, perhaps the prior exposure to simulation has a stronger impact on their perception of reality in simulation, than the design of this simulation itself?

We anticipated that the moulage groups would act quicker than the control in the time-to-action index. This was not supported by the data—in fact, in some instances the time-to-action was slower in the HighAuth group. The HighAuth group took (on average) 245.10 s to call for help, approximately 30 s longer than the control group and 50 s longer than LowAuth. Although the results were not significant, we hypothesise that HighAuth had more visual items to prioritise and consider as a part of their assessment process. Interestingly, the control group exposed the abdomen quicker than LowAuth (around 40 s difference), and HighAuth was very similar to the control group timing (3 s longer). It is plausible that this also is due to cue processing and the focus on audible cues may have prioritised their clinical decisions. In the interviews, participants identified they focused on certain verbal cues more than others as they believed perhaps there was hidden meaning in them or the confederate was trying to direct them a certain way. This does not explain why the HighAuth group exposed the abdomen so quickly, perhaps the visual cues on the face and arms may have triggered a need to investigate, demonstrating the effects of physical and semantic realism. Alternatively, maybe they found the authentic moulage distracting; however, this would appear unlikely given the participants’ discussion in interviews where they expressed the positive views towards moulage being included and the sense of urgency when it was present (demonstrating phenomenal realism).

The HighAuth group administered intravenous fluids slower (at least 50 s slower) than the LowAuth and control groups—again, this might be attributed to the number of visual cues that needed processing, signalling their active engagement with the simulation. These results differ from Mills et al. (2018) where they found in a comparison of moulage versus no moulage, that the paramedicine students in a moulage group were quicker to respond in time-to-treat [10]. Although there were differences in these times-to-treat, we do not interpret them as clinically significant. A 1-min difference in these items is unlikely to be life-threatening.

Interestingly, the participants of the HighAuth group were more likely to complete neurovascular observations as compared to the other two groups (*p* = 0.05). However, LowAuth were more likely to complete an abdominal palpation (*p* = 0.03). The HighAuth group applied gloves more often as compared to the other groups combined. The trend to conduct an abdominal palpation continued in the combined LowAuth/control group (*p* = 0.02). We considered that in the case of the high-authenticity group completing neurovascular observations, this might have been due to the additional visual stimuli of blood that triggered the need (signalling adequate conceptual realism) to check pupillary response and other neurovascular indicators. As hypothesised early in regard to the abdominal palpation, we felt that the absence of other factors (such as lacerations and grazing), the participant focused on the visual and audio cues of the abdominal injury. It was unsurprising to us that the HighAuth were more likely to apply gloves, students expressed in the interviews “*oh I saw the blood and thought, I need to put gloves on*”; this could have interesting implications for the role of moulage in teaching the use of gloves and personal protective equipment (PPE).

In considering the comparisons of moulage versus no moulage (control versus LowAuth/HighAuth) and HighAuth versus LowAuth/control, it was interesting that the significant results existed in the latter comparison as opposed to the first. We interpret this to mean that high-authenticity moulage has a more directive effect than the low- or no-moulage conditions.

In rating the moulage authenticity, participants rated the moulage, or lack of moulage, accordingly. This confirmed the ratings from the other self-report. Students consistently rated the control group moulage as low authenticity, LowAuth as medium authenticity and HighAuth as high authenticity. There has been no previous exploration of moulage authenticity and participants’ interaction with varied levels of moulage.

### Implications for moulage use

Although moulage may not impact clinical decisions detrimentally, this might not be enough to consider that moulage is insignificant. As we have seen in the interviews, participants identified that they spent significant periods of time trying to determine the conditions of simulation. This is supported by work exploring the process of suspending disbelief (SOD) in nursing students whereby authors state “enhanced environmental fidelity promotes SOD” [25]. Expanding on this further, if the conditions of simulation are not consistent across all exposures in a curriculum, it seems that this has impact on their ability to suspend disbelief, spending more time on focusing on deciphering the relevance. The underlying message here is that consistency across simulations is key.

Beyond this, moulage might contribute as a visual cue more significantly than expected—as demonstrated by the participants’ use of gloves and the completion of neurovascular observations and the students’ views. Simulation provides an opportunity to rehearse clinical practice and develop the ability to manage complex situations. Students described not taking the simulation seriously or “faking” it; what is the implication for this in transferring learning? Although our primary focus on this study was the impact on engagement, there is a potential link here. If the lack of authenticity of moulage prompts participants to take shortcuts, then it is worth questioning if we are contributing to negative learning? What we mean by this is the inadvertent, incorrect messages that we send to participants. In this scenario, no or poorly authentic moulage reduced the likelihood of applying PPE, sending the message that gloves are unimportant, thereby leading to “habitual unsafe behaviour” as described by Weller et al. [26]. The broader result might be an artificial type of learning, which we feel the students alluded to in their comments on “*doing it for the sake of doing it*”. Another extension of this negative learning might be the example of the slower abdominal palpation in the high-authenticity group—by not exposing participants to real conditions distracting factors, we might be inadvertently training them to only look for the obvious. Creating an authentic environment is often limited by cost; however, we would argue that not taking full advantage of simulation (significant expenditure is already there) would be a missed opportunity for rehearsing clinical practice.

Although not generalisable for all situations, the moulage might be better off being authentic. Moulage added complexity to the scenario. Highly authentic moulage might provide more consistent performance behaviours—what are the implications of this for high-stakes assessment versus technical skills? Perhaps low-authenticity moulage is be more confusing than high-authenticity.

### Limitations

Despite repeated efforts to recruit participants, we had no success in recruiting the required number indicated by statistical power calculations. The study was advertised with many weeks in advance and delivered at alternate times that might be suitable for students study schedules, including extending the study for an additional year. The simulation centre was based on the same site where students attended classes and placements. This provides limitations for the interpretation of results—the data results may have been too low to detect sizes of effect. Despite this, we did achieve statistically significant results that seemed to be accompanied by an adequate effect size. We recognise the limitations of a single assessor to determine the clinical actions completed and the time-to-treat information. A more robust approach might have been to have two assessors to then confirm the reliability of the judgement. This limitation is also extended to the coding of the interviews—although Grounded Theory techniques do not typically use multiple coders, we did not utilise the whole breadth of Grounded Theory. In this instance, it may have strengthened the work by having a second coder. Unfortunately, time and budgetary restraints limited the feasibility of these approaches.

The type of scenario used could be a potential limitation. Namely, a trauma situation may have more weight on the importance of engagement, as opposed to, for example, a dermatology scenario. Conversely, the urgency of a trauma scenario may have enough impetus to engage participants regardless of the level of authenticity, whilst the authenticity of dermatology might be more important than the authenticity of a trauma simulation.

## Conclusions

Exploring engagement is an emerging topic in simulation, with new techniques for measurement becoming available. These methods might provide better guides for measuring engagement. Other areas of work that should be explored include investigating how the quality of previous simulations determine engagement with scenario, and how moulage influences on so-called negative learning and developing good clinical habits. Additionally, further work could be done to explore the relationship of authentic moulage and working memory or cognitive load. This work would be interesting if replicated in a different clinical environment—for example, obstetrics, and other emergency scenarios. And, finally, it would be beneficial to explore the impact of authentic moulage on fully qualified clinicians or in other health professions groups.

This study adds to our understanding of the role moulage can play in the participants engagement in simulation. Within the context of undergraduate medical students, the use of authentic moulage may provide more consistent patterns of engagement, as compared to no or poor-quality moulage in simulation. Additionally, moulage may provide a more realistic process of prioritising care, thereby contributing to deep learning. We suggest that the authenticity of moulage contributes to learner engagement by highlighting the importance of the activity, allowing them to fully rehearse an activity and minimise instances of determining what is real and what a condition of the simulated environment is.

## Supplementary information

**Additional file 1:.** Supplementary data file

**Additional file 2:.** Stimulated recall interview instructions

**Additional file 3:.** Participant survey on perceived engagement

## Data Availability

All data generated or analysed during this study are included in this published article and its supplementary information files. If the data is not available in the supplement, they are available from the corresponding author on reasonable request.

## Abbreviations

LowAuth: Low-authenticity moulage
HighAuth: High-authenticity moulage
ISRI: Immersion Scale Reporting Instrument
SR: Stimulated Recall
JSP: Jessica Stokes-Parish
BJ: Brian Jolly
RD: Robbert Duvivier
ED: Emergency Department
EEN: Endorsed Enrolled Nurse
MARS: Moulage Authenticity Rating Scale
INACSL: International Nursing Association for Clinical Simulation and Learning
ANOVA: One-way analysis of variance
SPSS: Statistical Software Package for Social Science
OSCE: Objective Structured Clinical Examination
PPE: Personal protective equipment
SOD: Suspension of disbelief
