# Supplementary material for: How does moulage contribute to medical students’ perceived engagement in simulation? A mixed-methods pilot study
Source: Adv Simul (Lond). 2020 Aug 26;5:23. doi: 10.1186/s41077-020-00142-0 (PMC7449038; doi:10.1186/s41077-020-00142-0)

**Appendix 1: Supplementary Data – Statistics**

**Clinical actions completed**

**Neurological Observations**

| **Crosstab** | | | | | | |
| --- | --- | --- | --- | --- | --- | --- |
|  | | | grouprecode | | | Total |
|  |  |  | 1.00 | 2.00 | 3.00 |  |
| neuroobs | no | Count | 4 | 12 | 6 | 22 |
|  |  | Expected Count | 6.2 | 8.9 | 6.9 | 22.0 |
|  |  | % within neuroobs | 18.2% | 54.5% | 27.3% | 100.0% |
|  |  | % within grouprecode | 44.4% | 92.3% | 60.0% | 68.8% |
|  |  | % of Total | 12.5% | 37.5% | 18.8% | 68.8% |
|  | yes | Count | 5 | 1 | 4 | 10 |
|  |  | Expected Count | 2.8 | 4.1 | 3.1 | 10.0 |
|  |  | % within neuroobs | 50.0% | 10.0% | 40.0% | 100.0% |
|  |  | % within grouprecode | 55.6% | 7.7% | 40.0% | 31.3% |
|  |  | % of Total | 15.6% | 3.1% | 12.5% | 31.3% |
| Total | | Count | 9 | 13 | 10 | 32 |
|  |  | Expected Count | 9.0 | 13.0 | 10.0 | 32.0 |
|  |  | % within neuroobs | 28.1% | 40.6% | 31.3% | 100.0% |
|  |  | % within grouprecode | 100.0% | 100.0% | 100.0% | 100.0% |
|  |  | % of Total | 28.1% | 40.6% | 31.3% | 100.0% |

| **Chi-Square Tests** | | | | | | |
| --- | --- | --- | --- | --- | --- | --- |
|  | Value | df | Asymptotic Significance (2-sided) | Exact Sig. (2-sided) | Exact Sig. (1-sided) | Point Probability |
| Pearson Chi-Square | 6.189^a^ | 2 | .045 | .043 |  |  |
| Likelihood Ratio | 6.873 | 2 | .032 | .053 |  |  |
| Fisher's Exact Test | 6.269 |  |  | .048 |  |  |
| Linear-by-Linear Association | .409^b^ | 1 | .522 | .629 | .347 | .157 |
| N of Valid Cases | 32 |  |  |  |  |  |
| a. 3 cells (50.0%) have expected count less than 5. The minimum expected count is 2.81. | | | | | | |
| b. The standardized statistic is -.640. | | | | | | |

| **Crosstab** | | | | | | |
| --- | --- | --- | --- | --- | --- | --- |
|  | | | grouprecode | | | Total |
|  |  |  | 1.00 | 2.00 | 3.00 |  |
| Abdopalp | no | Count | 1 | 0 | 4 | 5 |
|  |  | Expected Count | 1.4 | 2.0 | 1.6 | 5.0 |
|  |  | % within Abdopalp | 20.0% | 0.0% | 80.0% | 100.0% |
|  |  | % within grouprecode | 11.1% | 0.0% | 40.0% | 15.6% |
|  |  | % of Total | 3.1% | 0.0% | 12.5% | 15.6% |
|  | yes | Count | 8 | 13 | 6 | 27 |
|  |  | Expected Count | 7.6 | 11.0 | 8.4 | 27.0 |
|  |  | % within Abdopalp | 29.6% | 48.1% | 22.2% | 100.0% |
|  |  | % within grouprecode | 88.9% | 100.0% | 60.0% | 84.4% |
|  |  | % of Total | 25.0% | 40.6% | 18.8% | 84.4% |
| Total | | Count | 9 | 13 | 10 | 32 |
|  |  | Expected Count | 9.0 | 13.0 | 10.0 | 32.0 |
|  |  | % within Abdopalp | 28.1% | 40.6% | 31.3% | 100.0% |
|  |  | % within grouprecode | 100.0% | 100.0% | 100.0% | 100.0% |
|  |  | % of Total | 28.1% | 40.6% | 31.3% | 100.0% |

| **Chi-Square Tests** | | | | | | |
| --- | --- | --- | --- | --- | --- | --- |
|  | Value | df | Asymptotic Significance (2-sided) | Exact Sig. (2-sided) | Exact Sig. (1-sided) | Point Probability |
| Pearson Chi-Square | 7.053^a^ | 2 | .029 | .032 |  |  |
| Likelihood Ratio | 7.998 | 2 | .018 | .032 |  |  |
| Fisher's Exact Test | 6.115 |  |  | .032 |  |  |
| Linear-by-Linear Association | 3.133^b^ | 1 | .077 | .118 | .071 | .056 |
| N of Valid Cases | 32 |  |  |  |  |  |
| a. 3 cells (50.0%) have expected count less than 5. The minimum expected count is 1.41. | | | | | | |
| b. The standardized statistic is -1.770. | | | | | | |

High versus Low and Control

| **Crosstab** | | | | | |
| --- | --- | --- | --- | --- | --- |
|  | | | HvLC | | Total |
|  |  |  | h | lc |  |
| Abdopalp | no | Count | 4 | 1 | 5 |
|  |  | Expected Count | 1.6 | 3.4 | 5.0 |
|  |  | % within Abdopalp | 80.0% | 20.0% | 100.0% |
|  |  | % within HvLC | 40.0% | 4.5% | 15.6% |
|  |  | % of Total | 12.5% | 3.1% | 15.6% |
|  | yes | Count | 6 | 21 | 27 |
|  |  | Expected Count | 8.4 | 18.6 | 27.0 |
|  |  | % within Abdopalp | 22.2% | 77.8% | 100.0% |
|  |  | % within HvLC | 60.0% | 95.5% | 84.4% |
|  |  | % of Total | 18.8% | 65.6% | 84.4% |
| Total | | Count | 10 | 22 | 32 |
|  |  | Expected Count | 10.0 | 22.0 | 32.0 |
|  |  | % within Abdopalp | 31.3% | 68.8% | 100.0% |
|  |  | % within HvLC | 100.0% | 100.0% | 100.0% |
|  |  | % of Total | 31.3% | 68.8% | 100.0% |

| **Chi-Square Tests** | | | | | |
| --- | --- | --- | --- | --- | --- |
|  | Value | df | Asymptotic Significance (2-sided) | Exact Sig. (2-sided) | Exact Sig. (1-sided) |
| Pearson Chi-Square | 6.555^a^ | 1 | .010 | .024 | .024 |
| Continuity Correction^b^ | 4.142 | 1 | .042 |  |  |
| Likelihood Ratio | 6.141 | 1 | .013 | .024 | .024 |
| Fisher's Exact Test |  |  |  | .024 | .024 |
| N of Valid Cases | 32 |  |  |  |  |
| a. 2 cells (50.0%) have expected count less than 5. The minimum expected count is 1.56. | | | | | |
| b. Computed only for a 2x2 table | | | | | |

**Self-reported Engagement Data**

| **ANOVA** | | | | | | |
| --- | --- | --- | --- | --- | --- | --- |
|  | | Sum of Squares | df | Mean Square | F | Sig. |
| **I felt engaged in the simulation** | Between Groups | .724 | 2 | .362 | 1.059 | .359 |
|  | Within Groups | 10.246 | 30 | .342 |  |  |
|  | Total | 10.970 | 32 |  |  |  |
| **At no point did I disengage from the simulation** | Between Groups | 1.866 | 2 | .933 | 1.399 | .263 |
|  | Within Groups | 20.013 | 30 | .667 |  |  |
|  | Total | 21.879 | 32 |  |  |  |
| **The appearance of moulage contributed to my engagement in the simulation** | Between Groups | 3.081 | 2 | 1.540 | 1.939 | .161 |
|  | Within Groups | 23.829 | 30 | .794 |  |  |
|  | Total | 26.909 | 32 |  |  |  |
| **The appearance of moulage did not cause me to disengage in the simulation*** | Between Groups | 9.014 | 2 | 4.507 | 4.231 | .024 |
|  | Within Groups | 31.956 | 30 | 1.065 |  |  |
|  | Total | 40.970 | 32 |  |  |  |
| **The authenticity of moulage is important in simulation**. | Between Groups | 2.731 | 2 | 1.366 | 1.878 | .170 |
|  | Within Groups | 21.814 | 30 | .727 |  |  |
|  | Total | 24.545 | 32 |  |  |  |
| **This simulation was a realistic representation of a trauma scenario*** | Between Groups | 6.513 | 2 | 3.256 | 5.364 | .010 |
|  | Within Groups | 18.214 | 30 | .607 |  |  |
|  | Total | 24.727 | 32 |  |  |  |
| **The moulage used was a realistic representation of a trauma scenario*** | Between Groups | 15.310 | 2 | 7.655 | 11.067 | .000 |
|  | Within Groups | 20.751 | 30 | .692 |  |  |
|  | Total | 36.061 | 32 |  |  |  |
| **The trauma victim looked similar to a real trauma victim*** | Between Groups | 18.950 | 2 | 9.475 | 12.397 | .000 |
|  | Within Groups | 22.929 | 30 | .764 |  |  |
|  | Total | 41.879 | 32 |  |  |  |
| **The appearance of the simulator made me feel like I was in a real trauma situation*** | Between Groups | 5.343 | 2 | 2.672 | 3.869 | .032 |
|  | Within Groups | 20.717 | 30 | .691 |  |  |
|  | Total | 26.061 | 32 |  |  |  |
| **It was easy to treat the simulator as a trauma victim*** | Between Groups | 6.065 | 2 | 3.032 | 5.752 | .008 |
|  | Within Groups | 15.814 | 30 | .527 |  |  |
|  | Total | 21.879 | 32 |  |  |  |
| **The simulation compares favorably with other simulation experiences I have had*** | Between Groups | 3.130 | 2 | 1.565 | 3.306 | .050 |
|  | Within Groups | 14.203 | 30 | .473 |  |  |
|  | Total | 17.333 | 32 |  |  |  |
| **This simulation would offer a good learning opportunity for training and assessment of trauma** | Between Groups | 1.065 | 2 | .532 | 1.246 | .302 |
|  | Within Groups | 12.814 | 30 | .427 |  |  |
|  | Total | 13.879 | 32 |  |  |  |
| **The appearance of the victim contributed positively to the training experience*** | Between Groups | 5.430 | 2 | 2.715 | 3.792 | .034 |
|  | Within Groups | 21.479 | 30 | .716 |  |  |
|  | Total | 26.909 | 32 |  |  |  |

**Post-hoc tests for self-reported engagement**

| **Multiple Comparisons** | | | | | | | |
| --- | --- | --- | --- | --- | --- | --- | --- |
| Tukey HSD | | | | | | | |
| Dependent Variable | (I) Group | (J) Group | Mean Difference (I-J) | Std. Error | Sig. | 95% Confidence Interval | |
|  |  |  |  |  |  | Lower Bound | Upper Bound |
| **The appearance of moulage did not cause me to disengage in the simulation*** | 1 | 2 | .77778 | .44095 | .199 | -.3093 | 1.8648 |
|  |  | 3 | 1.37778^*^ | .47421 | .018 | .2087 | 2.5468 |
|  | 2 | 1 | -.77778 | .44095 | .199 | -1.8648 | .3093 |
|  |  | 3 | .60000 | .42732 | .352 | -.4535 | 1.6535 |
|  | 3 | 1 | -1.37778^*^ | .47421 | .018 | -2.5468 | -.2087 |
|  |  | 2 | -.60000 | .42732 | .352 | -1.6535 | .4535 |
| **This simulation was a realistic representation of a trauma scenario*** | 1 | 2 | .52381 | .33291 | .272 | -.2969 | 1.3445 |
|  |  | 3 | 1.16667^*^ | .35801 | .008 | .2841 | 2.0493 |
|  | 2 | 1 | -.52381 | .33291 | .272 | -1.3445 | .2969 |
|  |  | 3 | .64286 | .32262 | .131 | -.1525 | 1.4382 |
|  | 3 | 1 | -1.16667^*^ | .35801 | .008 | -2.0493 | -.2841 |
|  |  | 2 | -.64286 | .32262 | .131 | -1.4382 | .1525 |
| **The moulage used was a realistic representation of a trauma scenario*** | 1 | 2 | .62698 | .35533 | .199 | -.2490 | 1.5030 |
|  |  | 3 | 1.75556^*^ | .38213 | .000 | .8135 | 2.6976 |
|  | 2 | 1 | -.62698 | .35533 | .199 | -1.5030 | .2490 |
|  |  | 3 | 1.12857^*^ | .34435 | .007 | .2797 | 1.9775 |
|  | 3 | 1 | -1.75556^*^ | .38213 | .000 | -2.6976 | -.8135 |
|  |  | 2 | -1.12857^*^ | .34435 | .007 | -1.9775 | -.2797 |
| **The trauma victim looked similar to a real trauma victim*** | 1 | 2 | 1.07143^*^ | .37351 | .020 | .1506 | 1.9922 |
|  |  | 3 | 2.00000^*^ | .40168 | .000 | 1.0097 | 2.9903 |
|  | 2 | 1 | -1.07143^*^ | .37351 | .020 | -1.9922 | -.1506 |
|  |  | 3 | .92857^*^ | .36197 | .040 | .0362 | 1.8209 |
|  | 3 | 1 | -2.00000^*^ | .40168 | .000 | -2.9903 | -1.0097 |
|  |  | 2 | -.92857^*^ | .36197 | .040 | -1.8209 | -.0362 |
| **The appearance of the simulator made me feel like I was in a real trauma situation*** | 1 | 2 | .81746 | .35505 | .071 | -.0578 | 1.6927 |
|  |  | 3 | .98889^*^ | .38182 | .038 | .0476 | 1.9302 |
|  | 2 | 1 | -.81746 | .35505 | .071 | -1.6927 | .0578 |
|  |  | 3 | .17143 | .34407 | .873 | -.6768 | 1.0197 |
|  | 3 | 1 | -.98889^*^ | .38182 | .038 | -1.9302 | -.0476 |
|  |  | 2 | -.17143 | .34407 | .873 | -1.0197 | .6768 |
| **It was easy to treat the simulator as a trauma victim*** | 1 | 2 | .19048 | .31020 | .814 | -.5743 | .9552 |
|  |  | 3 | 1.03333^*^ | .33360 | .011 | .2109 | 1.8557 |
|  | 2 | 1 | -.19048 | .31020 | .814 | -.9552 | .5743 |
|  |  | 3 | .84286^*^ | .30061 | .023 | .1018 | 1.5839 |
|  | 3 | 1 | -1.03333^*^ | .33360 | .011 | -1.8557 | -.2109 |
|  |  | 2 | -.84286^*^ | .30061 | .023 | -1.5839 | -.1018 |
| **The simulation compares favorably with other simulation experiences I have had*** | 1 | 2 | .46825 | .29398 | .264 | -.2565 | 1.1930 |
|  |  | 3 | .81111^*^ | .31615 | .040 | .0317 | 1.5905 |
|  | 2 | 1 | -.46825 | .29398 | .264 | -1.1930 | .2565 |
|  |  | 3 | .34286 | .28489 | .460 | -.3595 | 1.0452 |
|  | 3 | 1 | -.81111^*^ | .31615 | .040 | -1.5905 | -.0317 |
|  |  | 2 | -.34286 | .28489 | .460 | -1.0452 | .3595 |
| **The appearance of the victim contributed positively to the training experience*** | 1 | 2 | .730 | .362 | .125 | -.16 | 1.62 |
|  |  | 3 | 1.044^*^ | .389 | .030 | .09 | 2.00 |
|  | 2 | 1 | -.730 | .362 | .125 | -1.62 | .16 |
|  |  | 3 | .314 | .350 | .646 | -.55 | 1.18 |
|  | 3 | 1 | -1.044^*^ | .389 | .030 | -2.00 | -.09 |
|  |  | 2 | -.314 | .350 | .646 | -1.18 | .55 |
| *. The mean difference is significant at the 0.05 level. | | | | | | | |

**Moulage Authenticity Rating Scale Results**

| **ANOVA** | | | | | |
| --- | --- | --- | --- | --- | --- |
| Physical | | | | | |
|  | Sum of Squares | df | Mean Square | F | Sig. |
| Between Groups | 195.261 | 2 | 97.631 | 10.232 | .000 |
| Within Groups | 276.708 | 29 | 9.542 |  |  |
| Total | 471.969 | 31 |  |  |  |

| **Multiple Comparisons** | | | | | | |
| --- | --- | --- | --- | --- | --- | --- |
| Dependent Variable: Physical | | | | | | |
| Tukey HSD | | | | | | |
| (I) Group | (J) Group | Mean Difference (I-J) | Std. Error | Sig. | 95% Confidence Interval | |
|  |  |  |  |  | Lower Bound | Upper Bound |
| 1 | 2 | -3.76923^*^ | 1.33946 | .023 | -7.0772 | -.4612 |
|  | 3 | -6.40000^*^ | 1.41928 | .000 | -9.9051 | -2.8949 |
| 2 | 1 | 3.76923^*^ | 1.33946 | .023 | .4612 | 7.0772 |
|  | 3 | -2.63077 | 1.29928 | .124 | -5.8395 | .5780 |
| 3 | 1 | 6.40000^*^ | 1.41928 | .000 | 2.8949 | 9.9051 |
|  | 2 | 2.63077 | 1.29928 | .124 | -.5780 | 5.8395 |
| *. The mean difference is significant at the 0.05 level. | | | | | | |

| **ANOVA** | | | | | |
| --- | --- | --- | --- | --- | --- |
| Cognitive | | | | | |
|  | Sum of Squares | df | Mean Square | F | Sig. |
| Between Groups | 168.211 | 2 | 84.105 | 8.718 | .001 |
| Within Groups | 279.758 | 29 | 9.647 |  |  |
| Total | 447.969 | 31 |  |  |  |

| **Multiple Comparisons** | | | | | | |
| --- | --- | --- | --- | --- | --- | --- |
| Dependent Variable: Cognitive | | | | | | |
| Tukey HSD | | | | | | |
| (I) Group | (J) Group | Mean Difference (I-J) | Std. Error | Sig. | 95% Confidence Interval | |
|  |  |  |  |  | Lower Bound | Upper Bound |
| 1 | 2 | -4.80342^*^ | 1.34682 | .004 | -8.1296 | -1.4772 |
|  | 3 | -5.41111^*^ | 1.42708 | .002 | -8.9355 | -1.8867 |
| 2 | 1 | 4.80342^*^ | 1.34682 | .004 | 1.4772 | 8.1296 |
|  | 3 | -.60769 | 1.30643 | .888 | -3.8341 | 2.6187 |
| 3 | 1 | 5.41111^*^ | 1.42708 | .002 | 1.8867 | 8.9355 |
|  | 2 | .60769 | 1.30643 | .888 | -2.6187 | 3.8341 |
| *. The mean difference is significant at the 0.05 level. | | | | | | |

| **Multiple Comparisons** | | | | | | |
| --- | --- | --- | --- | --- | --- | --- |
| Dependent Variable: Combined | | | | | | |
| Tukey HSD | | | | | | |
| (I) Group | (J) Group | Mean Difference (I-J) | Std. Error | Sig. | 95% Confidence Interval | |
|  |  |  |  |  | Lower Bound | Upper Bound |
| 1 | 2 | -8.57265^*^ | 2.39442 | .003 | -14.4860 | -2.6593 |
|  | 3 | -11.81111^*^ | 2.53709 | .000 | -18.0768 | -5.5454 |
| 2 | 1 | 8.57265^*^ | 2.39442 | .003 | 2.6593 | 14.4860 |
|  | 3 | -3.23846 | 2.32259 | .357 | -8.9745 | 2.4975 |
| 3 | 1 | 11.81111^*^ | 2.53709 | .000 | 5.5454 | 18.0768 |
|  | 2 | 3.23846 | 2.32259 | .357 | -2.4975 | 8.9745 |
| *. The mean difference is significant at the 0.05 level. | | | | | | |

| **ANOVA** | | | | | |
| --- | --- | --- | --- | --- | --- |
| OVERALL (Global rating) | | | | | |
|  | Sum of Squares | df | Mean Square | F | Sig. |
| Between Groups | 7.360 | 2 | 3.680 | 7.897 | .002 |
| Within Groups | 13.515 | 29 | .466 |  |  |
| Total | 20.875 | 31 |  |  |  |

| **Multiple Comparisons** | | | | | | |
| --- | --- | --- | --- | --- | --- | --- |
| Dependent Variable: OVERALL (Global rating) | | | | | | |
| Tukey HSD | | | | | | |
| (I) Group | (J) Group | Mean Difference (I-J) | Std. Error | Sig. | 95% Confidence Interval | |
|  |  |  |  |  | Lower Bound | Upper Bound |
| 1 | 2 | -.598 | .296 | .125 | -1.33 | .13 |
|  | 3 | -1.244^*^ | .314 | .001 | -2.02 | -.47 |
| 2 | 1 | .598 | .296 | .125 | -.13 | 1.33 |
|  | 3 | -.646 | .287 | .079 | -1.36 | .06 |
| 3 | 1 | 1.244^*^ | .314 | .001 | .47 | 2.02 |
|  | 2 | .646 | .287 | .079 | -.06 | 1.36 |
| *. The mean difference is significant at the 0.05 level. | | | | | | |

**ISRI – Scatterplot**

Legend: Group 1 (Control), Group 2 (LowAuth), Group 3 (HighAuth)


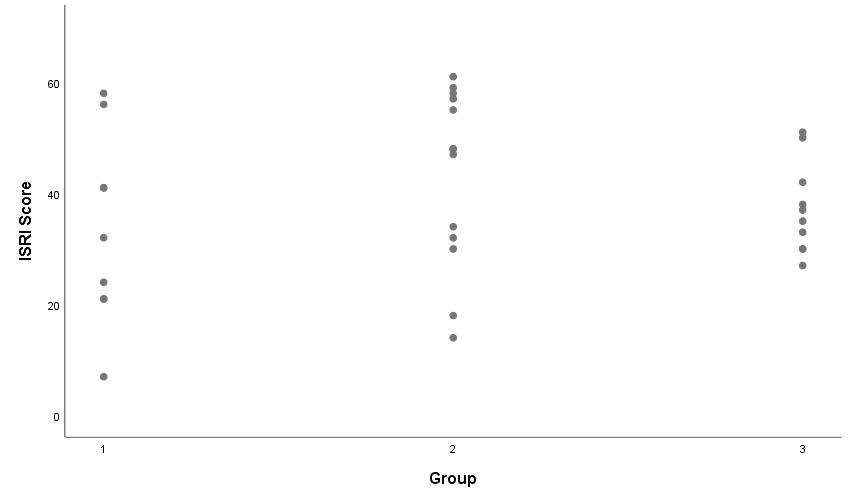

Supplement: Supplementary file 1 — Additional file 1:. Supplementary data file [file 41077_2020_142_MOESM1_ESM.docx]
