# Supplementary material for: How does moulage contribute to medical students’ perceived engagement in simulation? A mixed-methods pilot study
Source: Adv Simul (Lond). 2020 Aug 26;5:23. doi: 10.1186/s41077-020-00142-0 (PMC7449038; doi:10.1186/s41077-020-00142-0)
Supplement: Supplementary file 2 — Additional file 2:. Stimulated recall interview instructions [file 41077_2020_142_MOESM2_ESM.docx]

**Stimulated Recall Interview - Instruction for Interviewer**

**Duration: 40 mins**

The following sentence/paragraph is to be read to the participant at commencement of the Stimulated Recall Interview (SRI).

“We will now commence the Stimulated Recall Interview. I will play back the video of the simulation, and allow you to reflect on your engagement with the simulation. I have watched the video and made note of areas of interest (in engagement and disengagement, and related to moulage) that occurred in the simulation, and have some questions. I would like you to watch the video, and stop it at any point to describe what you were thinking regarding your engagement in the simulation/moulage. If you become too involved watching the video, I will stop the video at the pre-annotated points of relevance/interest and ask questions.”

Crafting questions:

Questions will be crafted based on your observation of the simulation activity. Using the Immersion Rating Scale as a guide, your questions should be focused on activities related to engagement, disengagement and moulage.

Do not ask questions about:

- Clinical decisions
- Teamwork
- Communication

Do ask questions about:

- Moulage (in general, its appearance, their interaction with moulage)
- Engaging in the activity or periods of disengagement

In general terms:

*What happened?*

*Why?*

*What did you think about…(example)?*

Example questions:

When you first saw the moulage/narrative of moulage, what did you think?

I noticed you did not address the moulage/narrative of moulage, what were you thinking about it?

When you assessed the wound (moulage), what did you think?
