# Supplementary material for: How does moulage contribute to medical students’ perceived engagement in simulation? A mixed-methods pilot study
Source: Adv Simul (Lond). 2020 Aug 26;5:23. doi: 10.1186/s41077-020-00142-0 (PMC7449038; doi:10.1186/s41077-020-00142-0)
Supplement: Supplementary file 3 — Additional file 3:. Participant survey on perceived engagement [file 41077_2020_142_MOESM3_ESM.docx]

Please rate your agreement with the following statements:

1. I felt engaged in the simulation
2. At no point did I disengage from the simulation
3. The appearance of moulage contributed to my engagement in the simulation
4. The appearance of moulage did not cause me to disengage in the simulation
5. The authenticity of moulage is important in simulation.
6. This simulation was a realistic representation of a trauma scenario
7. The moulage used was a realistic representation of a trauma scenario
8. The trauma victim looked similar to a real trauma victim
9. The appearance of the simulator made me feel like I was in a real trauma situation
10. It was easy to treat the simulator as a trauma victim
11. The simulation compares favorably with other simulation experiences I have had
12. This simulation would offer a good learning opportunity for training and assessment of
13. The appearance of the victim contributed positively to the training experience
